# The proximal humeral locking plate positioning to the pectoralis major tendon in achieving the proper calcar screw location: a cadaveric study

**DOI:** 10.1186/s13018-021-02892-7

**Published:** 2022-01-04

**Authors:** Nattha Kulkamthorn, Naruebade Rungrattanawilai, Thanakorn Tarunotai, Nantaphon Chuvetsereporn, Piyachat Chansela, Ong-art Phruetthiphat

**Affiliations:** 1 Orthopaedics Department, Phramongkutklao Hospital, 315 Ratchvidhi Road, Thung Phaya Thai, Ratchathewee, Bangkok, 10400 Thailand; 2 Department of Orthopaedic Surgery, Queen Sirikit Naval Hospital, Phlu Ta Luang, Thailand; 3 Department of Orthopaedic Surgery, Nopparatratchathani Hospital, Bangkok, Thailand; 4 Department of Orthopaedic Surgery, Faculty of Medicine, Burapha University, Saen Suk, Thailand; 5 Department of Anatomy, Phramongkutklao College of Medicine, Bangkok, Thailand

**Keywords:** Pectoralis Major Tendon, Elongated Combi-Hole, Calcar Screw, Distance, A Cadaveric Study

## Abstract

**Background:**

Proximal humeral fracture is the third most common of osteoporotic fracture. Most surgical cases were treated by fixation with anatomical locking plate system. The calcar screw plays a role in medial support and improving varus stability. Proximal humerus fracture in elderly patients are commonly seen with greater tuberosity (GT) fracture. The GT fragment is sometimes difficult to use as an anatomic landmark for proper plate and screw position. Therefore, the insertion of pectoralis major tendon (PMT) may be used as an alternative landmark for appropriate plate and calcar screw position. The purpose of study is going to identify the vertical distance from PMT to a definite point on the position of locking plate.

**Methods:**

30 cadaveric shoulders at the department of clinical anatomy were performed. Shoulders with osteoarthritic change (*n* = 5) were excluded. Finally, 25 soft cadaveric shoulders were recruited in this study. The PHILOS™ plate was placed 2 mm posterior to the bicipital groove. A humeral head (HH) was cut in the coronal plane at the level of the anterior border of the PHILOS plate with a saw. A calcar screw was inserted close to the inferior cortex of HH. Distance from the upper border of elongated combi-hole (UB-ECH) to the upper border of pectoralis major tendon (UB-PMT) was measured. The plate was then moved superiorly until the calcar screw was 12 mm superior to the inferior border of HH and the distance was repeatedly measured.

**Results:**

The range of distance from UB-PMT to the UB-ECH was from − 4.50 ± 7.95 mm to 6.62 ± 7.53 mm, when calcar screw was close to inferior border of HH and when the calcar screw was 12 mm superior to the inferior border of HH, respectively. The highest probability of calcar screw in proper location was 72% when UB-ECH was 3 mm above UB-PMT.

**Discussion and conclusion:**

The GT fragment is sometimes difficult to use as an anatomic landmark for proper plate and screw position. PMT can be used as an alternative anatomic reference. UB-PMT can serve as a guide for proper calcar screw insertion. UB-ECH should be 3 mm above UB-PMT and three-fourths of cases achieved proper calcar screw location.

## Background

Proximal humeral fractures are the third most common osteoporotic fracture [1–3], after wrist and hip fractures. The incidence was 114 and 47 per 100,000 person-years among females and males, respectively [1]. Nonoperative treatment can be performed in most patients [1, 2], while some patients need surgical fixation. Common implants used for fixation include the locking plate and intramedullary nail [2]. However, plate fixation is the most common surgical option for this fracture type [1, 2].

Anatomical reduction is important for a successful outcome after surgical fixation [4], but the varus angulation can be occurred after surgery [5]. Varus collapse is the second most common complication after plate fixation [6, 7]. The strength of the implant is essential to maintain the alignment of the fixation until bone healing occurs. New designs of locking plates and screws have been developed for osteoporotic fracture including treatment of elderly proximal humerus fracture. Although the locking plate provides more stability than the previously designed plate, the calcar screw has shown to be of significance because it can be inserted into the thinnest cortical bone, at the posterior and medial areas of the proximal humerus [8, 9]. The calcar screw plays a role in medial support and improving varus stability [10]. Proximal humerus fracture in elderly patients are commonly seen with greater tuberosity (GT) fracture. The GT fragment is sometimes difficult to use as an anatomic landmark for proper plate and screw position. Therefore, the insertion of pectoralis major tendon (PMT) may be used as an alternative landmark for appropriate plate and calcar screw position. The purpose of study is going to identify the vertical distance from PMT to a definite point on the position of locking plate to adjust the proper position for calcar screw insertion in proximal humerus fracture.

## Methods

After the Institutional Review Board (IRBRTA 2050/2561) approval, a study of 30 cadaveric shoulders at the Department of Clinical Anatomy, Phramongkutklao Hospital and College of Medicine, Bangkok, Thailand was performed between June 2018 and August 2018. Fifteen paired shoulders (*n* = 30) were included. Shoulders with osteoarthritic change (*n* = 5) were excluded. Finally, 25 soft cadaveric shoulders were recruited in this study. The cadaveric shoulders of 14 men (56%) and 11 women (44%) with normal upper limb and shoulder profile, with a mean age of 72.1 ± 13.9 years as shown in Table 1, were carefully dissected by a single surgeon to evaluate humeral head, greater tuberosity (GT), lesser tuberosity (LT), humeral shaft, and pectoralis major tendon (PMT).

**Table 1:** Demographic data (*n* = 25)

Variables	Statistics data
*Sex*	
Female	11 (44%)
Male	14 (56%)
*Age (years)*	
Mean ± SD	72.1 ± 13.88
Range	[42–92]
*Side*	
Left	12 (48%)
Right	13 (52%)

**Fig. 1:**
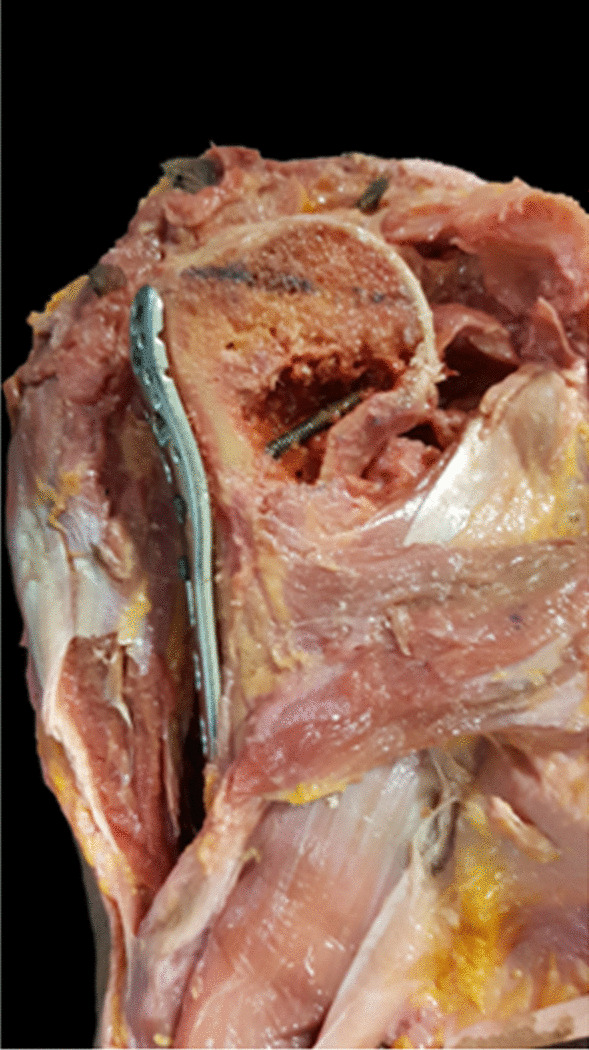
Calcar screw was placed closely to the inferior border of the humeral head

**Fig. 2:**
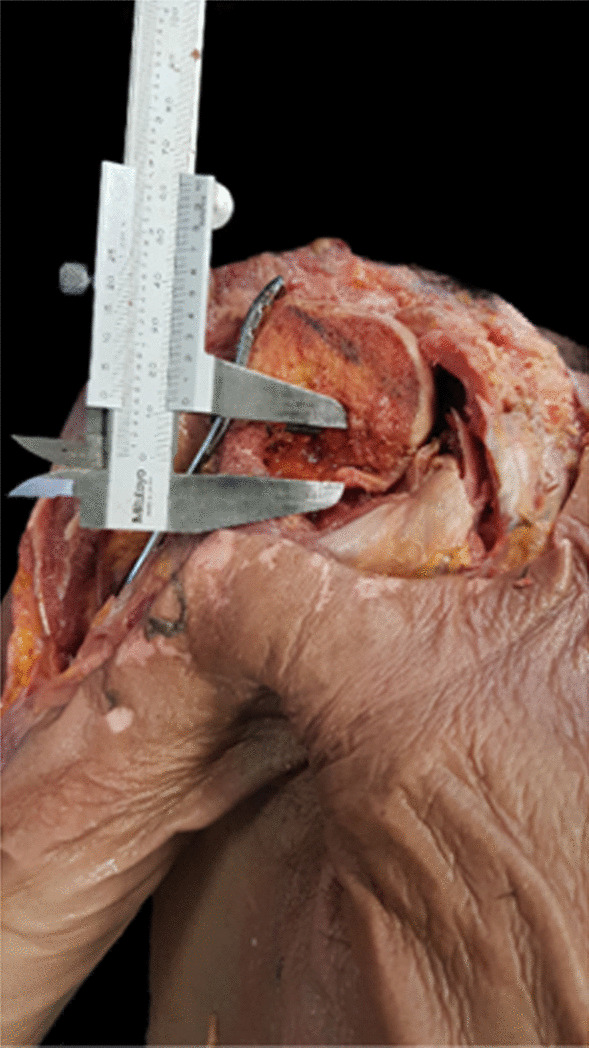
The calcar screw was placed 12 mm above the inferior border of the humeral head

The skin, subcutaneous tissue and deltoid muscle were removed. PMT insertion remained attached to the humeral shaft. All rotator cuff muscles were peeled-off close to GT and LT. The long head of the biceps brachii was then removed, and the length and thickness of PMT insertion were measured. Distance from the humeral head to GT and upper border of pectoralis major tendon (UB-PMT) insertion were measured using a level line perpendicular to the humerus axis. Three-hole PHILOS™ plate was placed 2 mm (mm) posterior to the bicipital groove followed by using a 3.5 mm cortical screw was to fix the plate to the humerus. The humeral head was cut in the coronal plane at the level of the anterior border of plate by electric oscillating saw. This cutting surface allowed for visualizing the calcar screw in the humeral head. The plate was then adjusted in the vertical plane until insertion of the calcar screw closed to the inferior cortex of the humeral head (Fig. 1). Distance from the upper border of elongated combi-hole (UB-ECH) to the upper border of pectoralis major tendon (UB-PMT) was measured. The plate was then moved upward until inserting the calcar screw located 12 mm superior to the inferior cortex of the humeral head (Fig. 2). Distance from UB-ECH to UB-PMT was then measured again. All parameters were performed three times by three different physicians and the averages were calculated as shown in Table 2. Vernier calipers with a reading error of 0.02 mm were used to measure. When UB-ECH was lower than UB-PMT, distances were presented with a minus.

**Table 2:** The association of anatomical landmarks of Pectoralis Major Tendon and PHILOS™ plate

Parameters	Mean ± SD (mm)	Minimum–Maximum (mm)
1.Total length of PMT	60.73 ± 7.89	47.29–77.16
1.1 Length of clavicular head of PMT	45.79 ± 7.94	35.44–59.17
1.2 Length of sternal head of PMT	15.00 ± 4.89	5.88–27.01
2.Thickness of PMT	2.26 ± 0.88	0.97–4.69
3.Distance from humeral head to tip of GT	7.84 ± 1.67	4.95–11.72
4.Distance from humeral head to UB-PMT	61.32 ± 11.23	37.74–83.71
5.Distance from UB-ECH to UB-PMT		
5.1 when calcar screw closed to inferior cortex of humeral head	− 4.50 ± 7.95	− 14.38–19.71
5.2 when calcar screw was inserted 12 mm above inferior cortex of the humeral head	6.62 ± 7.53	− 5.40–27.47

*PMT* Pectoralis Major Tendon, *GT* Greater tuberosity, *UB-ECH* Upper border of Elongated Combi-hole of PHILOS™ plate, *UB-PMT* Upper border of Pectoralis Major Tendon;

− (minus) when UB-ECH was lower than UB-PMT, distances were presented with a minus

### Data analysis

The data were collected and calculated using mean and standard deviation values including minimum and maximum to analyze data of all parameters (age, gender, length and thickness of PMT, distance from humeral head to tip of GT, distance from humeral head to UB-PMT, and distance from UB-ECH to UB-PMT).

## Results

**Fig. 3:**
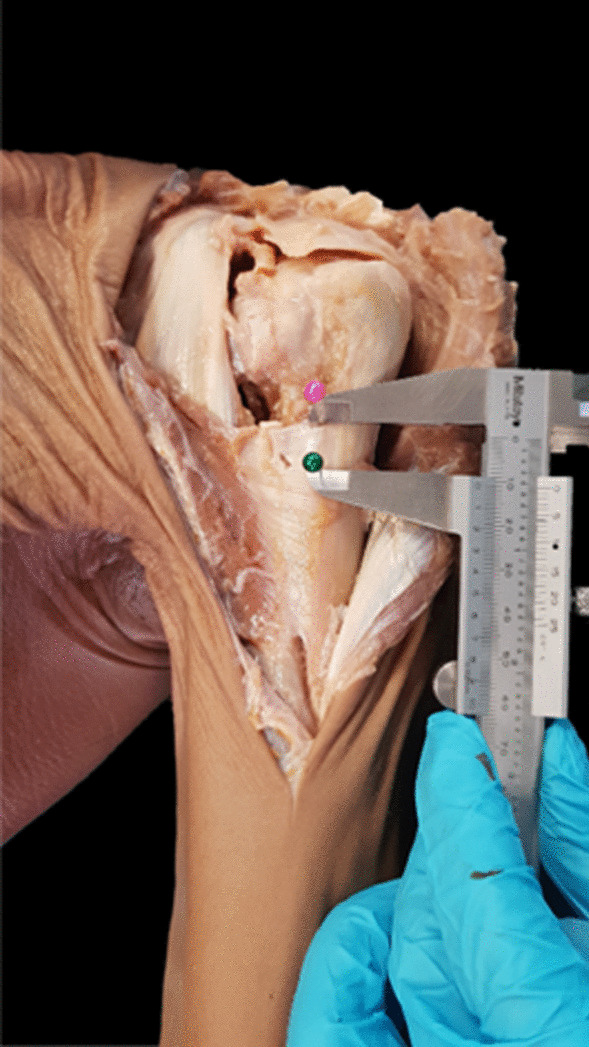
The measurement of extension of the pectoralis major tendon (PMT) insertion. The pink pin is at the upper border of the sternal head of PMT, while the green pin is at the upper border of the clavicular head of PMT

**Fig. 4:**
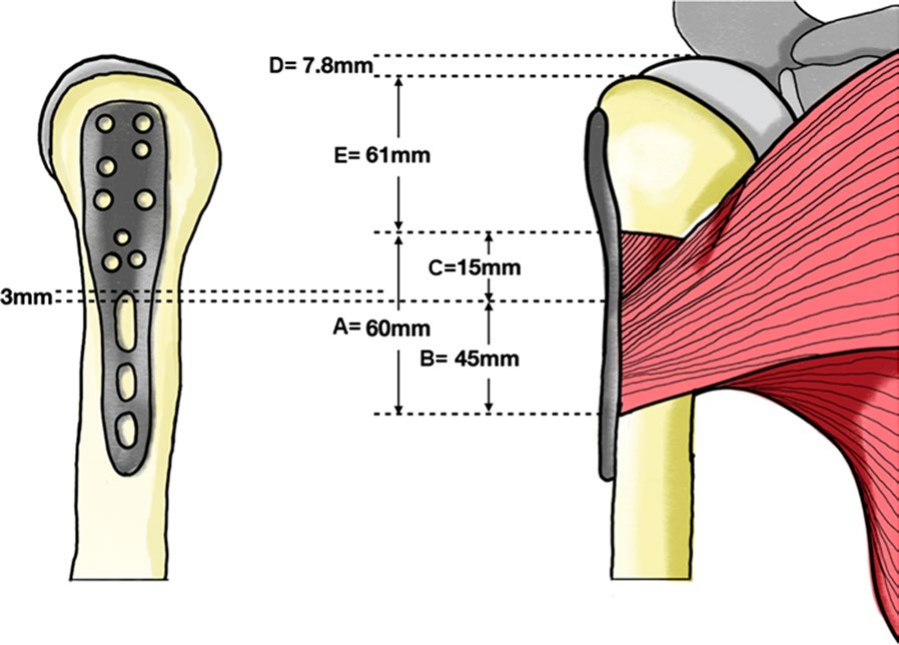
The anterior view of right proximal humerus and the mean distance of all anatomic parameters: A is total length of PMT insertion, B is length of clavicular head insertion, C is length of sternal head insertion, D is a distance from the top of the humeral head to tip of greater tuberosity (GT), and E is the distance from the tip of greater tuberosity to UB-PMT insertion

The origin of pectoralis major consists of three parts (the clavicular part, sternal part and abdominal part). However, the insertion of abdominal part is too small and difficult to identify. Therefore, we focus on dissection of the clavicular and sternal part in the actual clinical setting (Fig. 3). The sternal head was deeper and inserted higher than the clavicular head. The clavicular head was more clearly identified because its straight line was easily seen at the upper border of the muscle, while the sternal head was more difficult to identify. Thus, the clavicular head was used as the landmark of interest in this study and represented the PMT insertion later. The anatomic measurement of PMT and humeral head is showed in Fig. 4 and Table 2. The mean total length of PMT insertion (letter A of Fig. 4) was 60.73 ± 7.89 mm (47.29–77.16 mm). The mean length of clavicular head insertion (B of Fig. 4) was 45.79 ± 7.94 mm (35.44–59.17 mm), and mean length of the sternal head insertion was 15.00 ± 4.89 mm (5.88–27.01 mm) (C of Fig. 4). The mean thickness of PMT insertion was 2.26 ± 0.88 mm (0.97–4.69 mm). Distance from the top of the humeral head to tip of GT (D of Fig. 4) was 7.84 ± 1.67 mm (4.95–11.72 mm), and distance from the top of the humeral head to UB-PMT insertion (letter E of Fig. 4) was 61.32 ± 11.23 mm (37.74–83.71 mm).

**Fig. 5:**
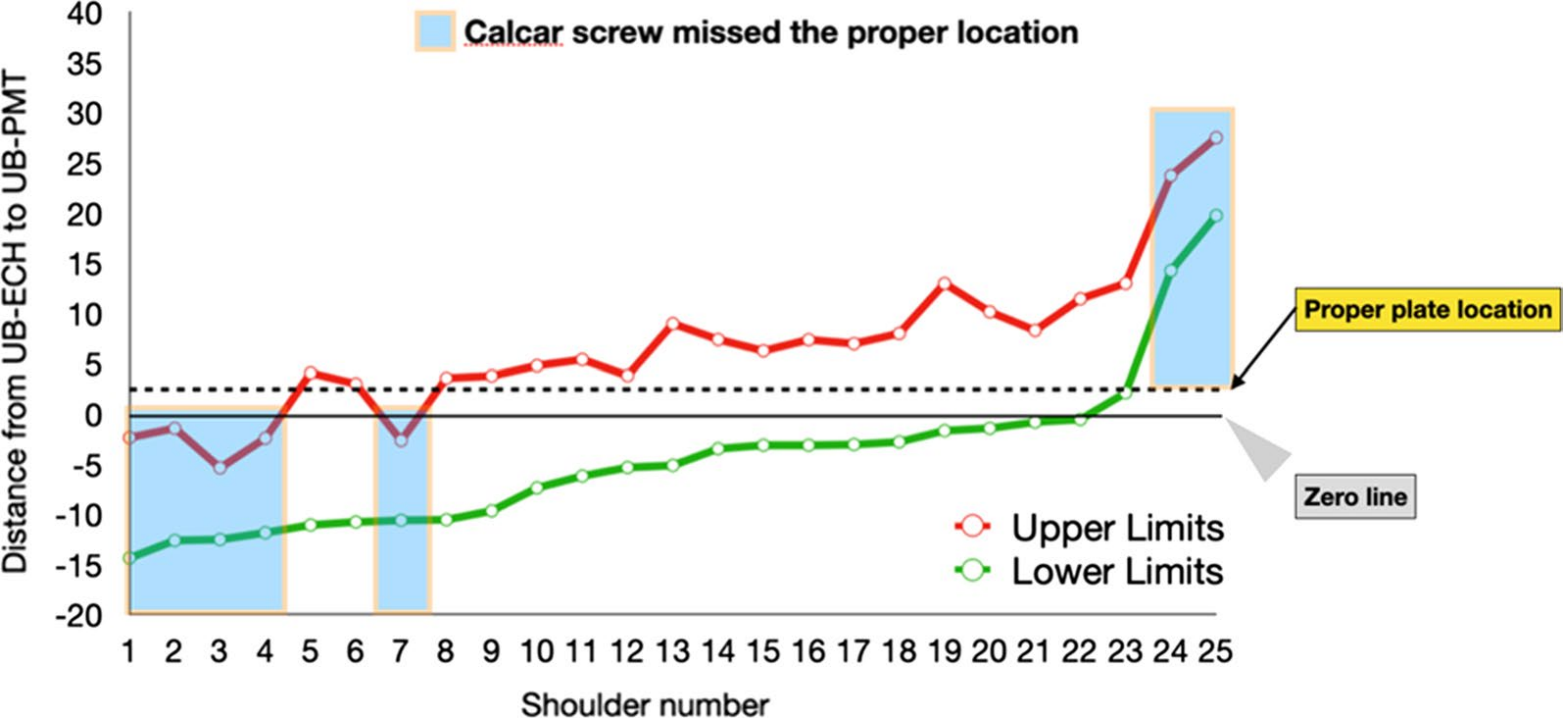
Upper and lower limits of the distance from the UB-ECH to UB-PMT. The green line indicates the distance when the calcar screw was inserted closely to the inferior border of the humeral head. The red line indicates the distance when the calcar screw was inserted 12 mm above the inferior border of the humeral head. Zero line means that UB-ECH was the same level as UB-PMT. When UB-ECH was lower than UB-PMT, distances were presented with a minus. Ideal plate position (Dashed line) should be placed 3 mm above the inferior border of the humeral head

Distance from UB-ECH to UB-PMT was − 4.50 ± 7.95 mm (− 14.38–19.71 mm) when the calcar screw was inserted closely to the inferior cortex of the humeral head; however, the distance was 6.62 ± 7.53 mm (− 5.40–27.47 mm) when the calcar screw was inserted 12 mm above the inferior cortex of the humeral head (Table 2). Upper and lower limits of the distance from UB-ECH to UB-PMT were plotted in a graph as shown in Fig. 5. The highest probability of proper calcar screw insertion was 18 out of 25 shoulders (72%) when the distance from UB-ECH to UB-PMT was 3 mm (dot line shown in Fig. 5).

## Discussion

New designs of locking plates and screws have been developed for osteoporotic fracture including treatment of elderly with proximal humerus fracture. They provide more stability than the previously designed plate because the calcar screw can be inserted into the thinnest cortical bone, at the posteromedial region of the proximal humerus [8, 9] to achieve medial support and to improve varus stability [10]. Proximal humerus fracture in elderly patients are often seen with GT fracture, and the GT fragment is sometimes difficult to use as an anatomic landmark for proper plate and screw position. Therefore, humeral shaft with the insertion of PMT may be used as an alternative landmark for appropriate plate and calcar screw position. The purpose of this study is going to identify the vertical distance from the PMT insertion to definite point on the locking plate for pointing out the proper position of the PHILOS™ plate on the proximal humerus.

Previous studies demonstrated normal anatomy of PMT without mention their two separated insertions [11–17]. This cadaveric study described both clavicular and sternal heads of PMT. The total length of PMT insertion of this study including clavicular and sternal heads was about 6 cm, slightly shorter than 7 cm in related studies [12, 17]. Thickness of PMT insertion was 2.2 mm, nearly similar to one related study at 1.4 mm [17]. Distance from the top of the humeral head to the upper border of PMT insertion was about 6 cm, which was comparable to previous studies (5.5 cm) [12–15]. Identifying both sternal and clavicular heads of PMT was practical for clinical application to point out the appropriate locking plate position.

This study showed that the UB-ECH was almost the same level as the UB-PMT insertion. As shown in Fig. 5, 23 out of 50 dots were above the zero line and 27 from 50 dots were below zero line. These results showed that PMT insertion was effortless to use as a clinical landmark for proper the PHILOS™ plate position.

The surgical technique reported by the AO Foundation described the fixation step starting with placing the plate to proximal fragments. The tip of the plate was recommended to be placed between 5 to 8 mm below the tip of GT [18]. The misplacement of the calcar screw in its appropriate location may occur and the orthopedic surgeon has to consume more time with the removal of all proximal locking screws to change the proper plate and calcar screw location. Thereby, initial plate placement on the proximal humerus using PMT insertion as the landmark is practical. An orthopedic surgeon can easily adjust the plate location upwardly or downwardly by inserting conventional screws in the ECH and the ideal calcar screw position was inserted into posteromedial region of the proximal humerus. With proper placement of the calcar screw, the proximal locking screws were then inserted as the final step to avoid any unnecessary bone void at the humeral head.

When UB-ECH was 3 mm higher than UB-PMT, the probability of obtaining proper calcar screw position was 72%. Most imperfect cases (28%) were composed of those with too high plate placement (20%) and those with too low plate position (8%). These missing cases were shown as blue boxes in Fig. 5. According to these data, a conventional screw should be inserted closely to the inferior border of ECH in order to have enough space to adjust the plate downwardly when the plate position is too high.

### Using the PMT insertion as an anatomical landmark for proper PHILOS™ plate position in clinical setting

**Fig. 6:**
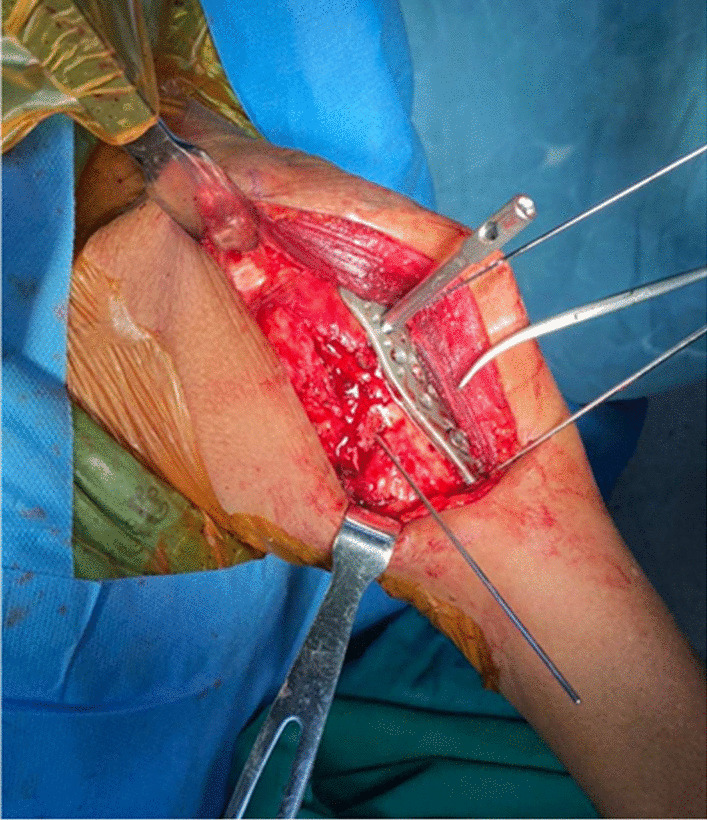
The PHILOSTM plate was placed on the lateral aspect of humerus by using two important landmarks: the PMT insertion for the vertical plane (superior-inferior alignment) while the bicipital groove for the horizontal plane (anterior–posterior alignment)

**Fig. 7:**
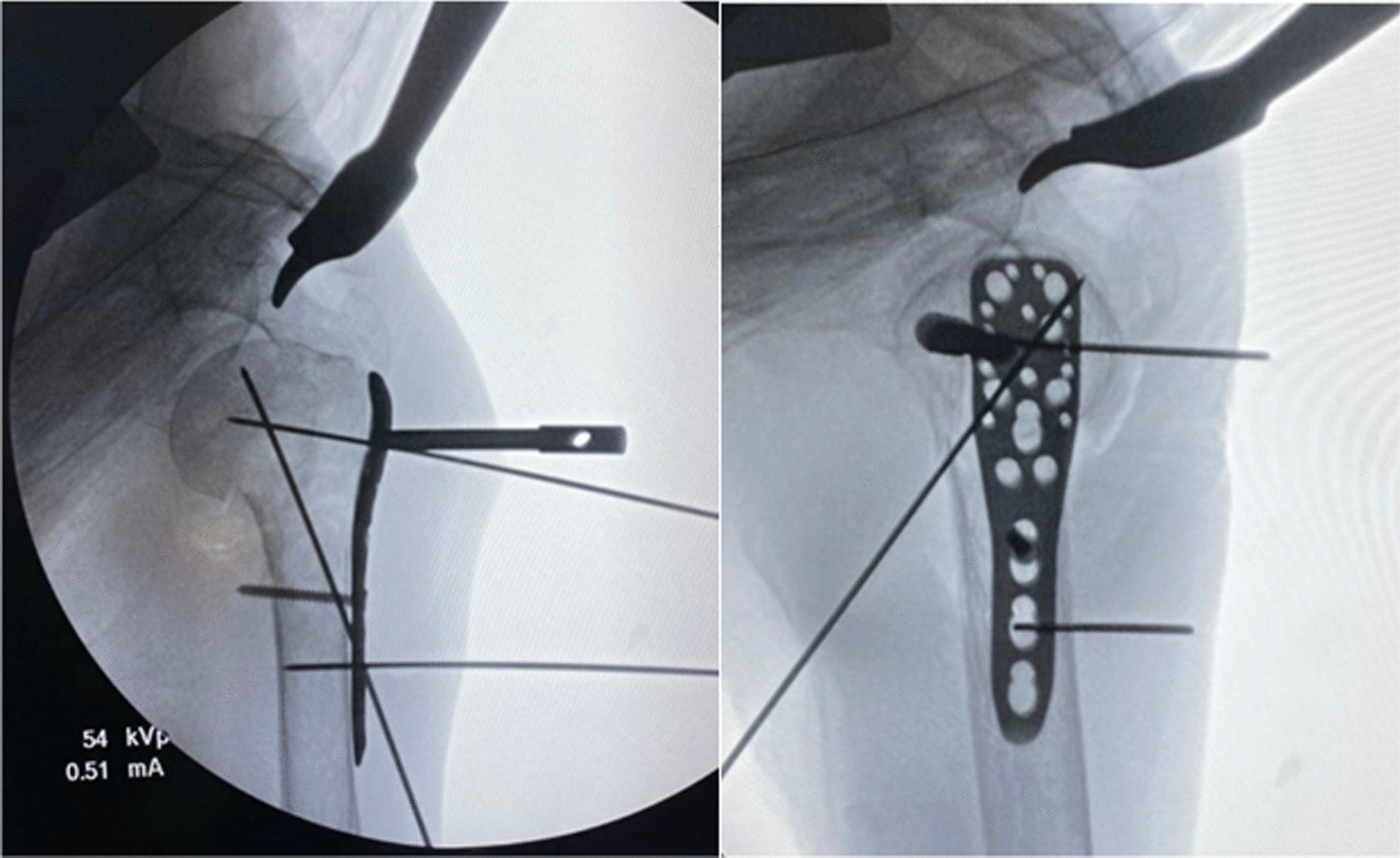
The cortical screw was applied into the ECH, and cortical screw should be inserted closely to the inferior border of ECH in order to have enough space to adjust the plate downwardly. The alignment of plate on humerus was then checked in both anterior–posterior and lateral views by fluoroscopy

**Fig. 8:**
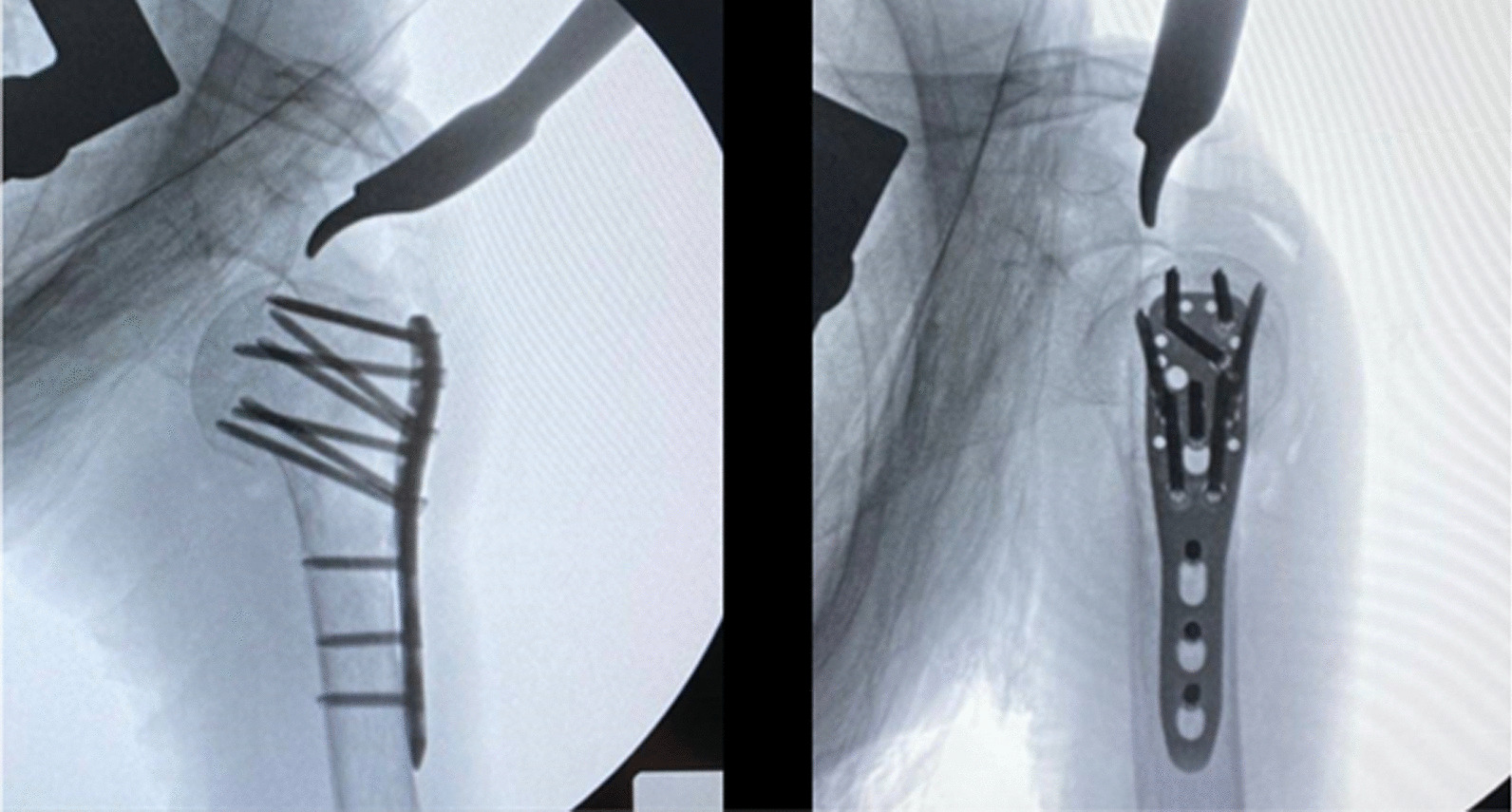
The calcar screws were in the proper location, the seven proximal locking screws were inserted into humeral head and the three distal locking screws were then applied

Patient was prepared on the operative table with semi beach chair position. Deltopectoral approach was performed to identify the proximal humerus fracture, greater tuberosity, lesser tuberosity, humeral head, humeral shaft, biceps tendon, and the PMT insertion. Open reduction and temporary fixation with multiple K-wires were done. PHILOS™ plate was placed on the lateral aspect of humerus by using two important landmarks (Fig. 6): the PMT insertion for the vertical plane (superior-inferior alignment) while the bicipital groove for the horizontal plane (anterior–posterior alignment). This plate was temporarily stabilized with two K-wires. The cortical screw was further applied into the ECH, and cortical screw should be inserted closely to the inferior border of ECH in order to have enough space to adjust the plate downwardly. The alignment of plate on humerus was checked in both anterior–posterior and lateral views by fluoroscopy (Fig. 7). Typically, two calcar screws were automatically inserted into the inferior border of humeral head with the proper position following the PMT insertion landmark. However, the position of proper calcar screw was checked again by fluoroscope. If the calcar screws were in the proper location, then seven proximal locking screws were inserted into humeral head and the three distal locking screws were then applied (Fig. 8).

This study should be interpreted in the context of a number of potential strengths and weaknesses. The strength of this study was used many fresh cadavers for identifying the anatomical landmark for proper proximal locking plate position and this preliminary study was effortless and practical for orthopedic surgeon to apply this in clinical setting. There were, however, a few limitations as following: (1) the patients in this study were Thai (Asian population), who differed from other group in terms of race and body frame. As a result, before applying this anatomical landmark to other populations, it should be validated; (2) There was no long-term clinical and radiographic outcome of surgical fixation for proximal humerus fracture using this alternative technique because of the pilot study. Therefore, further radiographic and clinical study is mandatory to identify functional outcome and complication; and (3) no evaluation of the height of the plate during different positions that concern the acromion with the PMT in this cadaveric study. In addition, if the calcar screw was inserted 12 mm above the inferior cortex of the humeral head, the upper border of the proximal humeral locking plate can impinge the acromion during shoulder abduction/forward flexion in the actual clinical setting.

## Conclusion

UB-PMT can serve as a guide for proper calcar screw insertion. UB-ECH should be 3 mm above UB-PMT and three-fourths of cases achieved proper calcar screw location. Although one fourth of cases missed, most involved placing the plate too high.

## Data Availability

The datasets used and/or analyzed during the current study are available from the corresponding author on reasonable request.

## Abbreviations

GT: Greater tuberosity
LT: Lesser tuberosity
PMT: Pectoralis major tendon
HH: Humeral head
UB-ECH: Upper border of elongated combi-hole
UB-PMT: Upper border of pectoralis major tendon
